# Round the Hospitals

**Published:** 1920-03-20

**Authors:** 


### ROUND THE HOSPITALS.

The King held an Investiture on March 10 at Buckingham Palace, and personally conferred the following Orders and decorations on members of the nursing services as follows:—Dame Commander of the Most Excellent Order of the British Empire (Military Division), Dame Sarah Oram, Q.A.I.M.N.S. This lady had also the honour of receiving the Bar to the Royal Red Cross. Royal Red Cross, First Class: Miss Catherine Renwick and Miss Isobel Whyte, Q.A.I.M.N.S.; Miss Mary Barrett, C.N.S.; Miss Ada White, T.F.N.S. Royal Red Cross, Second Class: Miss Alice Chirnside, Q.A.R.N.N.S.; Miss Maude Wilkin, Q.A.I.M.N.S.; Misses Loretto Fogarty, Florence Nice, Alice O'Connell, Thirza Robbins, Muriel Rowe, Jeanie Simon, Adela Stones, and Lilian

**ROUND THE HOSPITALS**—(*continued*).

Wass, Q.A.I.M.N.S.R.; Misses Louisa Finlayson, Annabella McLeod, Annie McMillan, and Margaret Moody, T.F.N.S.; Misses Margaret Ballana, Theresa King, and Bridget Stevin, St. John's Ambulance Brigade; Mrs. Kathleen Ross, South African M.N.S.; Misses Winifred Beausire, Annie Brawn, Dorothy Lawson, Alice Taylor, Edith Usher, and Edith Weston, V.A.D. Queen Alexandra received at Marlborough House the above members of the nursing service after the Investiture, together with Miss A. B. Smith, R.R.C., Matron-in-Chief.

At the Investiture held by the King at Buckingham Palace on March 12, His Majesty personally decorated members of the nursing services as follows:—Bar to the Royal Red Cross: Miss Annie Baird, Q.A.I.M.N.S. (R.); Miss Margaret Alexander, C.N.S. Royal Red Cross, 1st Class: Miss Ellen Tate and Miss K. Fraser-Wood, Q.A.I.M.N.S. (R.); Miss V. Spencer-Jones, C.N.S.. Royal Red Cross, 2nd Class: The Hon. E. Littleton and Miss C. Parke, Q.A.I.M.N.S.; Misses V. Blatchford, E. Brown, E. Chambers, F. Denton, S. Hall, E. Mackenzie, D. Maltby, Q. McAllan, and H. McIlwain, Q.A.I.M.N.S. (R.); Misses A. Bennett, B. Chadwick, K. Davies, and A. Hemmen, T.F.N.S.; Misses M. Andrews, A. Cross, E. Day, E. Swinton, Mrs. Millicent Battrum, Mrs. Eleanor Chisholm, C.N.S.; Misses F. Brunker, E. Coates, E. Davidson, D. Edwards, C. Leedham-Fuller, R. Gunn, M. Johnson, Mrs. Jane Mackay and Mrs. Leah Milner, B.R.C.S.; Miss Ethel Maxwell, St. John's A.B.; Miss E. Knox and Mrs. Katherine Marden-Ranger, Civil and War Hospitals; Misses C. Adamson, M. Adamson, M. Addenbrooke, L. Allen, M. Blake, and Mrs. Rose Cox, V.A.D. Queen Alexandra received all the above members of the nursing services, together with Miss A. B. Smith, R.R.C., Matron-in-Chief, at Marlborough House after the Investiture.

The Queen paid what may be called a valuable visit, on March 13, to No. 194 Queen's Gate, one of four hostels set on foot to relieve the anxieties of women demobilised from various branches of war work who urgently need temporary accommodation on their way through London. The Queen, who was presented with a handsome bouquet, inspected the house and was much pleased with it. Nearly all the forty ladies who are now in residence were present, as well as the superintendents from all the hostels, whom she complimented on their success in organisation. Some 6,000 women have been accommodated for varying periods; the homes are always full and have to turn away on an average 130 applicants a week. The Queen gave great pleasure to all by remaining to tea, when several of those present were presented to her, among others the matron of the Australian nurses and members of the V.A.D., Q.M.A.A.C., and the Women's Legion. A committee meeting was held afterwards, when Her Majesty took part in a long discussion as to the best steps to take to increase the available accommodation in view of the fact that the hostels are now self-supporting, and that girls of the educated class compelled to earn their living in London are experiencing great difficulties in getting housed.

The Queen's Fund, which helps to maintain Queen Victoria's Jubilee Institute for Nurses, raised nearly £8,000 last year for this most valuable national work, but was still £4,000 behind the estimated requirements. Queen Alexandra's Committee, under the presidency of Lady Northcote, undertakes to provide £2,000 a year, but an extra £5,000 is needed this year. There was never a time when the country could so ill afford to lose or see diminished the work of the Jubilee Institute in training nurses for districts and maintaining a high standard. We believe the special services rendered by the Jubilee Institute to be very imperfectly understood by the general public. They need explanation and illustration which they rarely get, and the Committee should turn their attention to improving their publicity department, and, in particular, their bare and too-sternly official report.

The Royal Southern Hospital, Liverpool, has issued an appeal for £12,000 to defray the cost of an extension to the nurses' home. The building is now approaching completion, and will cost approximately £8,500; but the furnishing and other extensions, which will shortly be required, will cost about an extra £3,500. The new Home will accommodate twenty-one additional nurses, and the managers state that this increase of staff will enable them to effect long-delayed reforms by lessening the hours of duty and giving one day off in seven. The appeal is headed by an earnest plea for justice to nurses, which exposes the fact that "the salary and conditions of life of the professional nurse have been such as to bear very unfavourable comparison with that of other classes of female labour. Her remuneration was less than that of the average domestic servant, her hours of duty were longer, and her time for holiday rest and recreation were not at all commensurate with the arduous duties she is called upon to perform." This frank statement, issued authoritatively by one of the leading boards of voluntary institutions in the country, cannot fail to have an appreciable effect on public opinion. Liverpool has always shown itself generous to nursing movements, and we have every hope of being able to announce shortly that the required sum has been subscribed.

London and its nursing sisters owe the fine Edith Cavell monument unveiled on March 17, which we illustrate on another page, to the inspiration of Viscount Burnham and the *Daily Telegraph*. The same public which is now generously contributing funds for the endowment of the College of Nursing was appealed to, not in vain, by this great daily paper to found a fitting memorial to one of the greatest nurses the profession has produced. In the splendid ceremony

**ROUND THE HOSPITALS**—*(continued).*

which crowned this appeal last Wednesday we see an augury of the complete success which will attend the efforts now in progress to secure the future of nurses from want and develop the profession on lines which shall reinforce its value to the public. We value the consistent respect, we might almost say reverence, which the *Daily Telegraph* accords to the nursing profession even more than the substantial response it can elicit for objects touching the welfare of nurses. The public has known too little in the past of the aims, the sacrifices and national work of nursing sisters. Individually they shrink from publicity in the ordinary sense, and are not prone to give interviews or advertise themselves. But they do value from their hearts the cordial sympathy and honour accorded to them impersonally in the pages of this leading paper which is noted for its advocacy of many a noble cause and has never, perhaps, pleaded more persuasively and with greater compelling power than for the nurses.

In response to a request made by several members of the Association of Hospital Matrons, its annual meeting has been postponed this year from April to June. It will be held at St. Thomas's Hospital on June 19. Meanwhile the next quarterly meeting will be held, by the kind invitation of Miss Watt, R.R.C., at the Radcliffe Infirmary, Oxford, on May 1.

On the retirement of Miss Gertrude Payne, after upwards of twenty years' devoted service, from the matronship of the Hospital for Sick Children, Great Ormond Street, the Committee has elected as her successor Miss M. C. Tisdale, R.R.C., the present matron of Paddington Green Children's Hospital. Miss Tisdale, who was trained at King's College Hospital, has had a wide experience in the nursing world. She has been sister at Queen Mary's Hospital for Children, Carshalton; ward-sister and home-sister at The Queen's Hospital for Children, Hackney Road; night superintendent and assistant matron at St. Mary's Hospital, Paddington; sister at the 1st Eastern General Hospital, Cambridge; and matron of the 2nd London General Hospital, Chelsea. Such experience should prove of great advantage to the Hospital for Sick Children at the present interesting stage in its development, and cannot fail to ensure that in Miss Tisdale the hospital has a worthy successor to Miss Payne.

The many graduates of the training-school for nurses at the London Temperance Hospital will learn with deep regret that their matron, Miss A. I. Richardson, has retired after twenty-eight years' work there. No one who took part in the wonderful reunion of past and present nurses held in the O.P. Hall on the twenty-first anniversary of Miss Richardson's tenure of office will ever forget the manifestation of love and reverence shown to her, and the appreciation of the homelike atmosphere, characteristic of the London Temperance Hospital under her rule. She has been succeeded in the matronship by Miss M. S. Donaldson, late matron of Mount Vernon Hospital, Northwood. The London Temperance Hospital is also losing Sister Dora Hinton, who is giving up her work there after twenty-eight years' service. It is said that there is not a nurse who has been trained at the hospital during the last twenty-five years who does not feel that any success she may have attained in the nursing profession is largely due to the training she received under Sister Dora. Not only in the technical part of the work, but by the untiring devotion and care shown to every patient in her wards, her example has been an inspiration and lesson to every one of her nurses.

The Memorial to the members of Queen Alexandra's Imperial Military Nursing Service and its Reserve and of the Territorial Force Nursing Service whose lives were given in the war is to take the form of a monument on which their names will be inscribed, to be erected in London. This is in accord with the expressed desire of the majority of the subscribers. The Committee have suggested that the Scottish and Irish nurses should be specially commemorated in Scotland and Ireland by a Memorial Tablet, on which should be inscribed the names of those who gave their lives. It is proposed to place the Tablets in a suitable spot in Edinburgh and Dublin respectively. The amount now subscribed is over £1,700.

A slight alteration in the pattern of the uniform of Queen Alexandra's Imperial Military Nursing Service has been authorised for outdoor wear, a grey coat and skirt taking the place of the long coat hitherto worn. A white silk or cotton shirt with a grey crêpe-de-chine tie may also be worn with this coat and skirt.

Chelsea Infirmary is widely known as a training-school for nurses, and the probationers of the past and present who have learnt within its walls know well all they owe to their matron, Miss Eleanor C. Barton, whose forthcoming retirement is announced this week. Trained at the Royal Hants County Hospital, Winchester, and at St. Bartholomew's Hospital, Miss Barton first went to Chelsea as home superintendent, later becoming matron of the Westgate Convalescent Home for Children in connection with the Infirmary, and finally returning to Chelsea as matron. It is significant of the esteem in which Miss Barton is held that Miss Stansfield, late Chief Lady Inspector of the Local Government Board, once spoke of Miss Barton and "her genius of concentration," and it is this genius that has been of such value to the Poor-law nursing service and the nursing profession at large. A member of the original Council of the College of Nursing, she was re-elected with acclamation in 1918. Though her duties at Chelsea were arduous enough, she has always found time to attend meetings and functions of public nursing interest, and as President of the Poor-law Matrons' Association she has done much for the welfare of infirmary nurses. She was Principal Matron of the Third London General Hospital

**ROUND THE HOSPITALS—**(*continued*).

throughout the war, and was awarded the Royal Red Cross, First Class, in 1916. Miss Barton intends to live in London after her retirement in June, and hopes to continue to take an active interest in nursing matters.

Much is expected from the General Council of the League of Red Cross Societies, whose first conference at Geneva has now closed. It was attended by delegates from twenty-eight countries and formed undoubtedly the most powerful organisation for the relief of suffering which the world has ever seen. Mr. Balfour, President of the Council of the League of Nations, addressed an eloquent appeal to the Red Cross League to organise an effort worthy of its unique position for relieving the distress in Central and Eastern Europe, which "seems almost worse than war itself." In response the Conference passed a resolution to the effect that if the Governments will first furnish the essentials—food, clothing, and transport, needs too vast to be supplied from voluntary effort—the Red Cross League will at once make plans for relieving the distressed districts, and will appeal through the Red Cross Societies for doctors, nurses, medical supplies, invalid foodstuffs, and the necessary money. There are many nursing sisters eagerly awaiting news that their services can be utilised in combating the terrible suffering in the famine and typhus-stricken areas, and the time appears to be drawing near when they will be summoned.

The cowardly marauders who lately raided the Glenvera Private Hospital, Gort, at midnight and robbed seven nurses of money, watches, and trinkets, repented next day of their pitiful crime and sent back the stolen property. It may not prove so easy to efface from the minds of the insulted nurses the effect of being turned out of doors at midnight, lined up with their backs against a wall, and told they were to be shot. It is inconceivable there should be no clue to the perpetrators of this disgraceful drunken raid, which demands the severest penalty the law can inflict.

The Board of the Royal Infirmary, Sheffield, have decided to increase the salaries of the nursing staff as follows:—First year probationers from £13 to £18; second year from £18 to £22; third year from £23 to £30; fourth year from £33 to £40. The matron is prepared to receive applications from probationers who have had two years' training in military, fever, or special hospitals. Such candidates will be accepted for a three, instead of a four years' course.

A devoted partnership has come to a close at the Royal Alexandra Hospital, Rhyl, with the resignations of Miss Anson and Miss Bulkeley Johnson from the staff. In all Miss Anson has served the hospital for forty years, and for the past twelve she has been its lady superintendent. Miss Bulkeley Johnson's record is also lengthy; it extends to about thirty years, during the greater part of which she has assisted Miss Anson. The hospital committee has decided to give signal marks of its recognition to both. A ward will be named after Miss Anson, and the names of her and her colleague will be recorded on the hospital's Board of Benefactors.

Sister Peake, absent in France during the war, has returned to the matronship of the Harrow Cottage Hospital, which is resuming its normal activities. Only ten wounded soldiers were received last year, and the civilian patients numbered 150. The hospital has a nursing fund and employs three district nurses, whose salaries, we are glad to learn, have all been raised. The infant welfare centre has largely increased its work, and the Harrow Urban District Council have now taken over the maternity nursing and have appointed Nurse Kindell as their full-time maternity nurse. The managers of the hospital appeal for an increase of income to meet the increased expenditure. It is not very creditable to churchpeople in Harrow that the church collections have fallen off by £30.

It is announced in the *Spectator* that the Red Cross authorities have found, with much regret, that Major Keswick's beautiful house, Tyrrells Wood, with its magnificent grounds, is on too large a scale as regards accommodation to be used for the purposes of a V.A.D. convalescent home. Lady Ampthill now announces that the Red Cross has accepted the kind offer of Captain and Mrs. Fullerton James and Miss Hichens, of Beechgrove, Sunninghill, Berks, for the new home.

The sum of £1,250 was awarded in the Court of King's Bench, as damages for personal injuries in a suit brought by Miss Edith Spicer, a hospital nurse, against Miss Violet Hood, R.A.F. The circumstances were most unfortunate for Miss Spicer, who, while walking on the foot-path at Norbiton, was knocked down by an instruction motor-van driven by the defendant swerving to avoid a truck in the road. Miss Spicer sustained terrible injuries, both legs being broken. She said in her evidence that she had been for nineteen years a private and hospital nurse, and that her average income was £160 a year, though less at Addington Park War Hospital, to which she was attached at the time of the accident, in October 1918. She was taken to Kingston Hospital, and afterwards to St. Thomas's. Her special expenses afterwards she estimated at £198. Medical evidence was brought to show that she could never follow her profession again. The case turned on whether negligence could be proved against Miss Hood, and the legal point being very doubtful the Solicitor-General appealed to the Lord Chief Justice for his assistance in making a fair award to Miss Spicer, for whom the utmost sympathy was experienced. The Lord Chief Justice emphasised the fact that the compensation awarded would not be anything like as much as it would have been had the legal liability of the defendant been established, and the case was amicably settled as before stated.

